# Reducing vitamin D requests in a primary care cohort: a quality improvement study

**DOI:** 10.3399/bjgpopen20X101090

**Published:** 2020-11-04

**Authors:** Veena Patel, Clare Gillies, Prashanth Patel, Timothy Davies, Sajeda Hansdot, Virginia Lee, Mayur Lakhani, Kamlesh Khunti, Pankaj Gupta

**Affiliations:** 1 Department of Rheumatology, University Hospitals of Leicester NHS Trust, Leicester, UK; 2 Diabetes Research Centre, University of Leicester, Leicester General Hospital, Leicester, UK; 3 Leicester Diabetes Centre, Leicester General Hospital, Leicester, UK; 4 Department of Metabolic Medicine and Chemical Pathology, University Hospitals of Leicester NHS Trust, Leicester, UK; 5 NIHR Leicester Cardiovascular Biomedical Research Unit, Glenfield Hospital University Hospitals of Leicester NHS Trust, Leicester, UK; 6 West Leicestershire Clinical Commissioning Group, Leicester, UK

**Keywords:** vitamin D, general practice, cost savings, primary healthcare, general practitioners

## Abstract

**Background:**

Since 2000, vitamin D requests have increased 2–6 fold with no evidence of a corresponding improvement in the health of the population. The ease of vitamin D requesting may contribue to the rapid rise in its demand and, hence, pragmatic interventions to reduce vitamin D test ordering are warranted.

**Aim:**

To study the effect on vitamin D requests following a redesign of the electronic forms used in primary care. In addition, any potential harms were studied and the potential cost-savings associated with the intervention were evaluated.

**Design & setting:**

An interventional study took place within primary care across Leicestershire, England.

**Method:**

The intervention was a redesign of the electronic laboratory request form for primary care practitioners across the county. Data were collected on vitamin D requests for a 6-month period prior to the change (October 2016 to March 2017) and the corresponding 6-month period post-intervention (October 2017 to March 2018), data were also collected on vitamin D, calcium, and phosphate levels.

**Results:**

The number of requests for vitamin D decreased by 14 918 (36.2%) following the intervention. Changes in the median calcium and phosphate were not clinically significant. Cost-modelling suggested that if such an intervention was implemented across primary care in the UK, there would be a potential annual saving to the NHS of £38 712 606.

**Conclusion:**

A simple pragmatic redesign of the electronic request form for vitamin D test led to a significant reduction in vitamin D requests without any adverse effect on the quality of care.

## How this fits in

Vitamin D requests have increased 2–6 fold, which has led to a 3-fold increase in medical expenditure in the UK since 2000 onwards. It is possible that the ease of vitamin D test ordering may contribute to the rapid rise in its requesting. Thus, this quality improvement project was done to study the effect on vitamin D test ordering following a redesign of the electronic request forms. Implementation of a simple pragmatic redesign of the electronic request form led to a significant reduction in vitamin D requests by 14 918 (36.2%) without any adverse effects on the quality of care, with the potential annual saving to the NHS of £38 712 606. Redesigning the laboratory requesting forms in keeping with up-to-date evidence would help demand management for many other laboratory tests as noted in the literature. The study shows redesigning the request form led to the behavioural change in requesting vitamin D among primary physicians in the short term. Replication of the study on a larger scale and for a longer period with multi-level intervention (such as education for physicians and patients) would be needed to confirm the findings.

## Introduction

Globally, across varied healthcare systems, use of diagnostic tests is increasing steadily with the workload rising by 10% every year in the UK in the last two decades.^1–3^ Nevertheless, there has been a substantial increase in vitamin D testing and consequently prescriptions for low vitamin D across multiple countries, including Australia, Canada, France, Saudi Arabia, US, and the UK between 2006 and 2015.^4^ In the UK, during the last 15–18 years, there has been a 2–6 fold increase in vitamin D requests.^4–6^ The corresponding costs of analysis of vitamin D in primary care across the UK have increased 3-fold, with an estimated increase in annual spending of £28 million in 2004 to £76 million in 2011.^4^ This increase is without any clear gain in health benefits.^5^ Similar to the UK-wide trend of increase in vitamin D requests, Leicestershire with a population of 1.1 million people had 63 940 vitamin D requests in 2013, which rose by around two-thirds to 102 970 in 2017.

Vitamin D is linked with multiple conditions, including osteoporosis, autoimmune conditions, cardiovascular diseases, multiple sclerosis, maternity outcomes, cancer, and infection risk without proven causality.^4–11^ Furthermore, low vitamin D is present in a very large proportion of the population, raising the possibility that is this a normal variant.^12^ In addition, there is an increased awareness among medical professionals and the public about vitamin D, although there is uncertainty in its diagnostic utility. The raised awareness and support by physicians to meet patients' expectations during consultations have contributed noticeably to the vitamin D requesting behaviour.^1,4–6,9^

National and international guidelines recommend vitamin D testing be performed only in those patients who present with symptoms such as bone pain, muscle weakness, symptoms suggestive of rickets, or those patients who have low serum calcium levels.^5,11^ Vitamin D measurement is deemed to be unnecessary for people on a long-term maintenance dose of vitamin D and it is recommended that vitamin D is supplemented in children and all adults (including people at high risk) throughout the year without the need for routine monitoring.^5,10,11^

Further, in order to limit an overutilisation of vitamin D requests, there are initiatives by the national healthcare systems in France and Ontaria, Canada, to limit the measurement to a subset of high-risk conditions.^13,14^ Deschasaux *et al* were able to achieve a reduction in the requests using a screening questionnaire to identify the high-risk individuals for vitamin D testing.^15^ Enhancing both patients’ and physicians’ knowledge through educational sessions and giving timely feedback via telephone and computer have also shown to have a positive impact.^15–17^ Harnessing technology, such as by using electronic clinical decision support tools and modifying the electronic medical record to avoid duplications, are some other techniques that have resulted in reduction in vitamin D test ordering.^18,19^

Laboratory requesting or ordering forms are methods of communication between laboratory physicians and clinicians. Regular reviews of laboratory forms have assisted clinicians to use the laboratory tests rationally and appropriately.^20^ A study by Zaat *et al* showed an 18% reduction in laboratory tests requests by restructuring the paper-test ordering forms among GPs.^21^ Bailey *et al* changed the pathology request forms by removing some of the tests from the forms used by the GP, which resulted in 20%–70% reduction in the unwanted requests and test.^22^ Further, Smellie in his review, showed that measures such as uncoupling the tests form request panels, cascade testing within the laboratory, restricting test availability in certain areas (emergency or GP practice), and time-determined gating of duplicate requests, all helped in the appropriate use of the laboratory tests.^20^

Wong *et al* showed that a combination of educational programmes and deletion of the laboratory tests on request forms resulted in a 60% reduction in use of thyroid function tests.^23^ Shalev *et al* concurred that changes in the laboratory request forms either by addition or deletion of the tests, influenced the physician test ordering practice.^24^ The principle of reviewing and restructuring the laboratory request forms was further supported by Martin *et al*. In addition, Neilson *et al* utilised the test ordering software technology, which resulted in tests being withdrawn or added from the request forms in laboratory tests ordering system.^25,26^ Thus, this quality improvement project was planned to study the effect on vitamin D requests with the intervention to redesign the laboratory electronic request forms.

### Aim

The aim of this study was to determine the impact on vitamin D test ordering across Leicestershire, following a redesign of the electronic request forms used in primary care. In addition, potential harms and cost-saving associated with the intervention were also studied.

## Method

### Design

The laboratory services of the Department of Chemical Pathology and Metabolic Diseases, University Hospitals of Leicester (UHL) NHS Trust, UK, caters to the entire population of Leicestershire of approximately 1.1 million. There are 139 general practices.

### Setting

All general practices use electronic laboratory requesting forms called ICE (Integrated Clinical Environment [Sunquest Information Systems]), which are centrally managed at UHL.

### Intervention

The intervention was introduced in the ICE forms on 30 September 2017. Pre-intervention vitamin D requesting was available for primary care practitioners on the first page of the ICE screen, as shown in the screenshot in supplementary Figure S1a (circled red). In discussion and agreement with the clinical commissioning group (CCG), accessibility of vitamin D requests was moved to the second page from the first page, along with other chemistry tests, as shown in the screen shots in supplementary data Figure S1b and S1c. Data on vitamin D requests were collated from interrogating the laboratory information system (i-Lab) between October 2017 and March 2018. In order to negate the impact of the seasonal variation in the vitamin D levels, data on vitamin D requests in the same months in the previous year (that is, from October 2016 to March 2017) were collected as a control.

### Measures

The primary measure was the difference in the number of vitamin D requests pre- and post-intervention. Along with demographic characteristics of age, sex, and ethnic group, corresponding serum calcium and phosphate values were collected for individual cases in both the control and intervention groups to study any detrimental effect from the change in requesting pattern of vitamin D.

### Statistical analysis

Patient characteristics were compared in the pre- and post-intervention period with χ^2^ tests for categorical variables, *t*-tests for normally distributed continuous variables, and the Wilcoxon rank sum test for non-parametric continuous variables.

Data on the reasons for the vitamin D requests were analysed in 500 random samples from each group. Random numbers were generated via Microsoft Excel 2016 and the first 500 numbers were collected in each group. The sample size was decided on a pragmatic basis and no formal statistical analysis was conducted to evaluate the same. The request reasons were coded by the research team into eight categories namely: (1) low vitamin D, calcium, or phosphate; (2) osteomalacia or rickets; (3) muscle pain; (4) osteoporosis or its treatment; (5) bone pain; (6) chronic kidney disease; (7) tired; and (8) other reasons or request reason not stated.

### Cost analysis

Cost analysis was carried out to assess the potential cost-savings of the intervention for the NHS. Cost of testing for vitamin D was obtained from the National Institute for Health and Care Excellence (NICE) economic evaluation published in 2014, which estimated the cost per vitamin D test to be £16.50.^27^ This estimate was adjusted to 2017 prices using the hospital and community health services inflation index and a value of £17.17 per vitamin D test was used in the analysis.^28^

## Results

### Primary outcome

**Figure 1.**
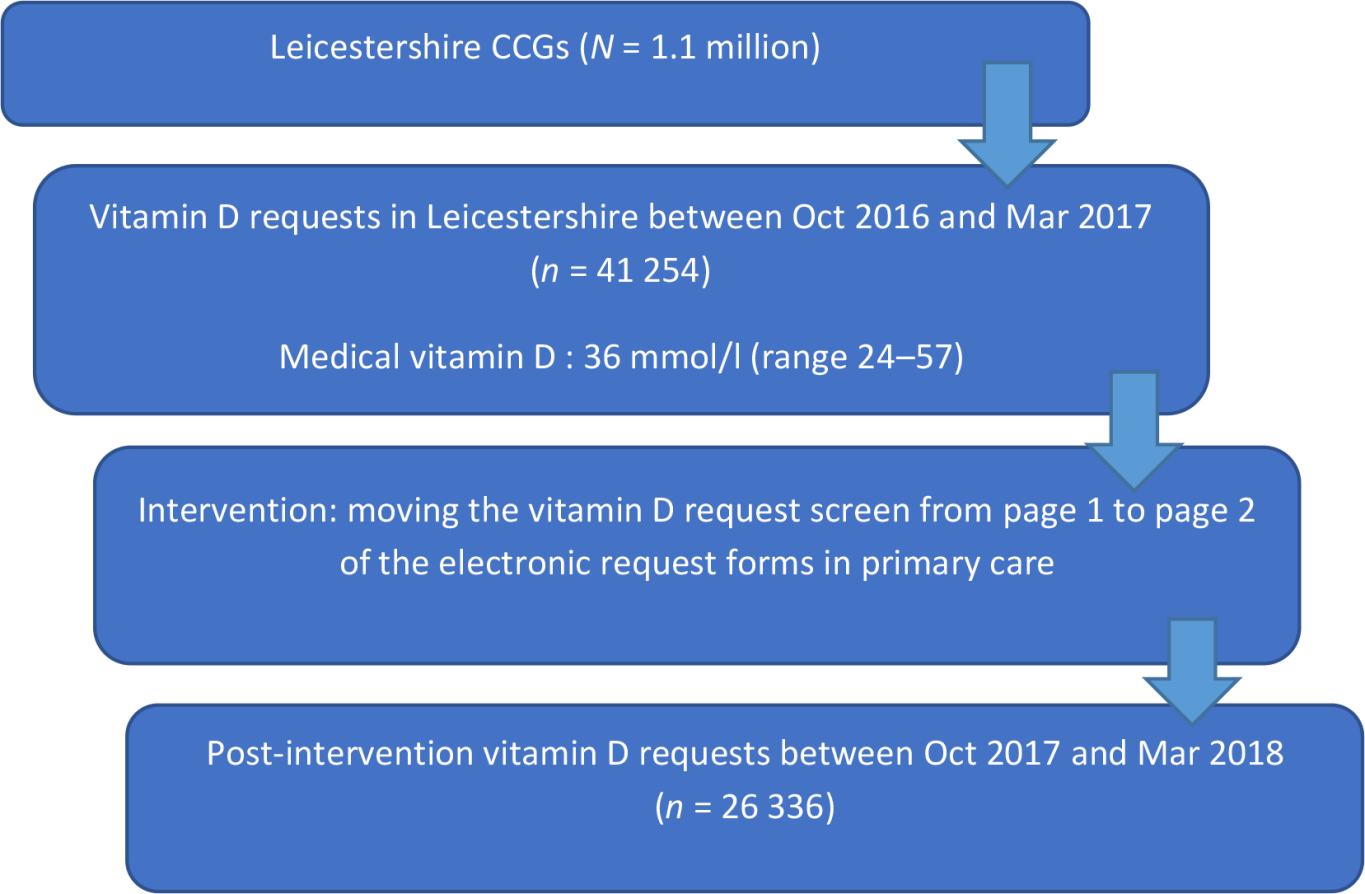
Vitamin D requests before and after the intervention

The number of requests for vitamin D decreased by 14 918 (36.2%) from 41 254 in the pre-intervention period to 26 336 in the post-intervention period (Table 1, Figure 1 and Supplementary Figure S2).

**Table 1.** Number of vitamin D requests, demographic characteristics, and biochemistry values in pre- and post-intervention groups

	**Pre-intervention (Oct 2016–Mar 2017**)	**Post-intervention (Oct 2017–Mar 2018**)	***P* value**
Total	41 254 (100)	26 336 (100)	<0.001
**Number of vitamin D requests**			
Requests with vitamin D <30 nmol/l	15 358 (39.4)	8810 (34.5)	<0.001
Requests with vitamin D 30–50 nmol/l	11 166 (28.6)	8044 (31.5)	<0.001
Requests with vitamin D >50 nmol/l	12 469 (32.0)	8671 (34.0)	<0.001
**Demographic characteristics**			
Age, years	47.9 (20.5)	46.5 (20.4)	<0.001
Male	13 914 (33.7)	8512 (32.3)	<0.001
*Ethnic* *group*			<0.001
Caucasian	20 103 (55.9)	11 991 (54.8)	
Asian	12 766 (35.5)	8327 (37.3)	
Black	1235 (3.4)	845 (3.8)	
Other	1890 (5.3)	1194 (5.3)	
**Biochemistry values**			
Vitamin D nmol/l^a^	36 (24–57)	38 (26–58)	<0.001
Calcium mmol/l	2.26 (0.09)	2.25 (0.09)	<0.001
Phosphate mmol/l^a^	1.11 (1.10, 1.11)	1.12 (0.99, 1.25)	<0.001

Values are *n* (%) or mean (standard deviation), apart from where indicated. Vitamin D levels were missing for 2261 requests made pre-intervention and 811 requests post-intervention. ^a^Median, interquartile range.

### Demographics

Percentages were calculated on the basis of number of participants with available data. Missing data were variable (age 0%, sex 2%, vitamin D 5%, ethnic group 14%, adjusted calcium 30%, and phosphate 31%).

### Statistical analysis

There was a reduction in requests in all of the three subgroups of vitamin D, that is: 'deficient' (30 nmol/l), 'levels may be inadequate in some groups' (30–50 nmol/l) and 'sufficient in most of the population' (>50 nmol/l) (Table 1 ).

The median vitamin D level in the pre-intervention group was 36 (IQR 24–57) mmol/l, and in the post-intervention levels was 38 (IQR 26–58) mmol/l (*P*<0.001) (Table 1, Supplementary Figure S3). As a proportion of total requests, the number of patients diagnosed with vitamin D <30 nmol/l reduced from 39.4% in the pre-intervention group to 34.5%.

The median serum calcium decreased by 0.01 mmol/l and the median serum phosphate increased by 0.01 mmol/l in the post- versus the pre-intervention groups (*P*<0.001 for both) (Table 1). The changes in these two parameters mimic the laboratory variation of these two parameters during the same period (Supplementary Figure S4). The pre- and post-intervention groups differed significantly statistically for ethnic group, age, and sex, with a greater proportion of males and Caucasians, with a slightly higher mean age, being referred for a test prior to the intervention.

Analysis of the reasons for the vitamin D requests showed that there were no changes in the requesting pattern of vitamin D in pre- and post-intervention groups (*P*<0.053). The maximum number of requests in each group was classified as 'other' (not related to vitamin D deficiency or treatment) or not stated on the form followed by tiredness (Supplementary Table S1).

### Cost analysis

The cost analysis estimated that a reduction of 36.2% (*n* = 14 918) in vitamin D requests would lead to an annual saving of £638 758 across Leicestershire (Supplementary Table S2).^27,28^ Assuming Leicester CCGs cover a population of 1 100 415, which constitutes 1.65% of the UK population of 66 579 184,^29^ the results can be extrapolated to estimate that delivering the intervention across primary care in the UK would lead to annual saving of £38 712 606 (Supplementary Table S2).^30^

## Discussion

### Summary

This study shows the impact of implementing a simple pragmatic redesign of the electronic request screens led to a substantial reduction in vitamin D requests by more than one-third. It reduced expenditure by more than half a million pounds in Leicestershire alone, which could translate to a potential saving of around £38 million if implemented across the UK.^30^

### Comparison with existing literature

The reduction in vitamin D requesting because of the change in the request screens is likely to be attributable to the change in the test ordering behaviour among GPs. This link between change in requesting behaviour and laboratory request forms has been previously illustrated in the literature.^21–25^ The present study also emphasises the need for a regular review of the laboratory forms to ensure the evidence-based, cost-effective use of NHS resources.^20,26^

The intervention addressing the laboratory forms is shown to be useful for the demand management in first 6 months in the study. Previous studies have emphasised on the multi-level intervention to bring a sustained behavioural change for effective demand management plans.^1,20,24,31,32^

Dissemination of the NICE guidelines would support clinicians to appropriately request the vitamin D test in the right group of patients with the ultimate goal of using the resourses judiciously to improve the quality of care. Primary care physicians are probably requesting vitamin D as this is associated with multiple conditions.^4^ It could also be to meet patients' expectations; that is, to find answers through diagnostic tests for all non-specific symptoms, such as generalised aches, pains, and tiredness. It could also be seen to be doing something to enhance patients' satisfaction to meet their needs during the consultation.^1,4,6^ Improving health literacy, promoting understanding of health through education and awareness, along with better communication strategies, would support the appropriate use of healthcare resources.^32^

### Strengths and limitations

This study has several strengths. It was a large study covering the entire Leicestershire population of approximately 1.1 million, with nearly 26 000 requests in the 6-month study period. The intervention was simple and easy to introduce, and can be implemented widely. The study was conducted anonymously, prospectively, and thus selection bias is limited. As the intervention was introduced across all Leicestershire GP practices, performance and exclusion bias are also minimised. Vitamin D requests have been collected from like months (that is, October 2016 to March 2017), adding to the strength of the study and counteracting the impact of the seasonal variation of vitamin D.

Some limitations are acknowledged in the study. The intervention was evaluated only for a short term and previous studies have demonstrated the need for multi-level intervention to bring a sustained behavioural change for effective demand management plans.^1,20,23,31,32^ Thus, replication of the study on a larger scale and for a longer period would be needed to confirm the findings. Further, the study was not designed to capture the reasons behind the requests for vitamin D. An understanding of the reasons and attitudes of practising physicians through qualitative studies would help in better understanding of the requesting behaviour of the physicians. Further, not all primary care practices across the UK have access to vitamin D on ICE in the same manner as in Leicestershire; for example, in Buckinghamshire primary care, vitamin D is not listed on the ICE forms, but has to be specifically requested on the order forms by physicians. Only a simple cost analysis was carried out for this study and a fully comprehensive health economic analysis was not performed. To truly understand the full cost implications of the intervention, a more comprehensive model incorporating a wider range of costs (such as medications following identification of low vitamin D levels, or complications when vitamin D levels aren’t addressed) and fixed cost analysis would need to be carried out. A caveat to consider is that changes in requesting volume are also dependent on overheads related to the cost of a test.^33^

### Implications for research and practice

The serum calcium, phosphate difference between the pre- and post-intervention group can be explained by corresponding laboratory variations. The median level of vitamin D in the pre-intervention and post-intervention groups was above the deficient range (vitamin D >30 nmol/l) and more than one-third had adequate vitamin D levels (above 50 nmol/l). There was also a decrease of around 5% in the total proportion of patients diagnosed with vitamin D deficiency (<30 nmol/l). Hence, it is summarised that the intervention did not lead to any significant clinical harm.

The implementation of a new, cost-neutral, simple, pragmatic redesign of the electronic request form for vitamin D measurement led to a 36.2% reduction in the tests requested in Leicestershire CCGs. Serum vitamin D, calcium, and phosphate levels are not adversely affected by this intervention. Redesigning the laboratory forms would help in demand management in the short term, but longer-term multicentre studies are needed to confirm these findings.
